# Seizures in children with dysembryoplastic neuroepithelial tumors of the brain—A review of surgical outcomes across several studies

**DOI:** 10.1007/s00381-015-2675-9

**Published:** 2015-03-21

**Authors:** Adrianna Ranger, David Diosy

**Affiliations:** 1 Department of Clinical Neurological Sciences, Division of Neurosurgery (Pediatric Neurosurgery), Schulich School of Medicine and Dentistry, Western University, London, Ontario Canada; 2 Department of Clinical Neurological Sciences, Division of Neurology (Epilepsy), Schulich School of Medicine and Dentistry, Western University, London, Ontario Canada; 3 Children’s Hospital, London Health Sciences Center, 800 Commissioners Road East, Room B1-138, London, Ontario N6A 4G9 Canada

**Keywords:** DNET, Pediatric brain tumors, Seizure surgery, Epilepsy

## Abstract

**Purpose:**

In children and adolescents, dysembryoplastic neuroepithelial tumors (DNETs) of the brain present with seizures almost 100 % of the time, potentially creating significant long-term morbidity and disability despite the generally indolent course of the lesion. These tumors also tend to be quite resistant to anti-epileptic drugs which, themselves, can be associated with long-term side effects and resultant disability. Many clinicians advocate early surgical resection of these lesions, but how effective this approach is, and how aggressive tumor removal should be, continues to be debated.

**Methods:**

We performed a systematic review of the relevant literature to identify all reports of DNET resections in pediatric patients published over the past 20 years. In all, over 3000 MEDLINE abstracts were reviewed, ultimately resulting in 13 studies with 185 pediatric DNET patients to review.

**Results:**

Surgical resection of the lesion was effective at improving seizures in over 98 % of patients and at achieving long-term seizure freedom in 86 %. Surgical resection of DNETs also appeared to be quite safe, with no reported perioperative deaths and an overall rate of postoperative complications of 12 %; the vast majority of these complications were transient.

**Conclusions:**

Total gross resection of the lesion was the only factor statistically correlated with long-term seizure freedom (*r* = 0.63, *p* = 0.03). However, data remain lacking regarding whether this translates into more extensive procedures—like brain mapping and partial lobectomies—being any more effective than simple lesionectomies alone. Further research is clearly needed to address this and other crucial questions.

## Introduction

In North America, no other solid tumor is as common or causes more deaths in children and adolescents than cancers of the brain [16, 26, 84], with roughly three quarters of patients presenting at less than 15 years of age [12]. The prognosis for long-term survival is much better in children than in adults, with up to half of pediatric brain tumor patients surviving long term [25]. The main reason for this enhanced survival is that children and adolescents tend to have lower-grade lesions [79, 95, 101]. Long-term survival is not without problems, however, with many pediatric brain tumor survivors continuing to suffer from significant morbidity [44, 63, 76, 90, 94, 108] and, sometimes, early death [90]. Among the more common long-term sequelae of brain tumors and their treatment in children are seizures, which can be quite disabling and, at times, life-threatening in themselves [90, 3, 56, 66, 67, 85, 87, 104, 106, 110]. In one study, seizures were the dominant predictor of disability in long-term brain tumor survivors [66, 67]. Seizures even increase a pediatric brain tumor survivor’s risk of suicide into adulthood [13]. Of the epileptogenic tumors, low-grade lesions comprise the majority [86]. Of these, dysembryoplastic neuroepithelial tumors (DNETs) present with seizures almost 100 % of the time [1, 65, 73, 97, 104].

Because these tumors typically are slow-growing and non-invasive, the primary objective of DNET management in most cases is control, if not eradication, of seizures. Because tumor-associated seizures tend to be more resistant to anti-epileptic drugs (AEDs) than idiopathic seizures [57, 98] and the long-term use of AEDs is not without significant risks in itself [22, 23, 56, 66, 68, 100, 107], including wide-ranging adverse effects on cognitive function and development [56, 100], this typically necessitates surgery to resect as much of the tumor as possible [21, 70, 73, 78, 89, 97, 104]. In addition, several studies have shown that radical removal of an epileptogenic brain tumor is a strong, and likely the strongest, predictor of seizure freedom [39]. On the other hand, especially among younger children, the risks of surgery cannot be discounted as, in addition to the risk of perioperative mortality, surgery also can adversely affect neurological development and function [38].

The primary purpose of this paper is to extensively and systematically review and analyze the literature on surgical outcomes in pediatric patients with DNETs, specifically looking at perioperative complications and mortality, short- and long-term seizure control, and short- and long-term survival.

## Search methods

The main search objective was to identify all papers involving pediatric patients with DNETs undergoing surgical resection over the past 20 years (1994–2014), in which the following information was available either for the entire sample or for individual patients (for papers in which patients with a range of tumor types are represented): number of patients with a DNET, (mean) age at the time of seizure onset, (mean) age at the time of surgery, age range, type of seizure, type of surgery, location of the lesion, number of subjects in which complete resection was achieved, number with immediate postoperative complications including recurrent/persistent seizures, (mean) length of follow-up, long-term survival, long-term neurological sequelae, the number with seizures at final follow-up, and final Engel rating [27]. The period 1994 to 2014 was selected because it was felt that older papers reported the results of studies with considerably less robust methodology and because most papers prior to 1995 did not report many of the specific variables of interest listed above.

All the included papers were identified via an extensive search of the PubMed database, using the following search terms: dysembryoplastic neuroepithelial tumor (*n* = 425), DNET (*n* = 193), neuroglial tumor (*n* = 401), neuroglial tumor and seizure (*n* = 1574), and neuroglial tumor and surgery (*n* = 871). Of these terms, only those with *n* ≤2000 were reviewed, because of the lack of specificity of longer lists. A total of 33 papers were identified in which surgery was conducted on children with DNETs, and the above-noted data were available in 24 [5, 9, 10, 15, 17, 21, 31, 35, 36, 47, 48, 51, 52, 54, 55, 58, 59, 70, 73, 75, 80, 89, 97, 105]. However, of these, only seven studies had pediatric DNET patients exclusively [9, 30, 58, 70, 73, 89, 97], two had both adult and pediatric patients, but individualized data [17, 59], and an additional four papers [5, 15, 47, 51] had individual data on DNET patients among patients with other lesions that allowed for the extraction of almost all of the variables of interest.

Analysis consisted of calculating means and percentages, Pearson correlation coefficients to identify the strength of correlation between study means of continuous variables, and likelihood ratios and Pearson *χ* ^2^ analyses to examine categorical variables. Where indicated, a *p* value of 0.05 or less was considered indicative of a statistically significant intergroup difference or correlation. Correlation strength was categorized as weak (*r* < 0.40), moderate (0.40–0.69), or strong (*r* ≥ 0.70) as indicated in the review of Taylor [102].

## Search results and analysis

Table 1 lists the 13 studies identified in which data of interest were available for patients with dysembryoplastic neuroepithelial tumors (DNETs), including six papers specific to pediatric DNET tumors for which totals and means are presented [9, 58, 70, 73, 89, 97] and seven papers with data presented for individual DNET patients from which totals and means could be calculated [5, 15, 17, 30, 47, 51, 58]. These 13 studies encompass 185 patients, of mean age 9.4 years, with individual patients ranging in age from 0.5 to 21 years. All the series were small, one paper reporting a single case [47] and only four having 20 or more patients [9, 58, 70, 74]. The largest study was that reported by Bilginer et al. with 29 pediatric DNET patients [9]. The mean sample size across the 13 series papers was 14.3. The mean duration of seizures prior to surgical resection of the underlying DNET across the 13 studies was 3.2 years but ranged widely from 4 and 7 months [15, 47] to 7 years [70, 97]. Complex partial seizures accounted for 86.4 % of seizures, ranging from 55.6 % [89] to 100 % [5, 30, 58]. The mean percentage of DNETs located within a temporal lobe across the 13 studies was 67.8 %, though this ranged quite broadly from 38.5 % [73] and 42.9 % [30] to 100 % [15]. Considering subjects individually, rather than assessing study means, the overall weighted percentage of patients presenting with complex partial seizures was 81.3 % and the percentage with a temporal lobe lesion 63.8 %. Gross total resection was achieved in 83.3 % of patients, the percentage lowered by a single study in which gross total resection was achieved in only 11 of 26 [73].

**Table 1:** Seizure response to surgical resection of dysembryoplastic neuroectodermal tumors

First author	Year published	No. of subjects	Mean age (year)	Age range	Surgical procedure	No. of total resection	% Total resection	Mean FU (month)	No. of seizure free	% Seizure free	No. of improved	% Improved	No. of perioperative deaths	No. of surgical complications
Babini	2013	4	12	5–18 years	L	?	?	102	3	75.0 %	4	100.0 %	0	0
Jo	2013	1	0.5	0.5 years	L	1	100.0 %	98	1	100.0 %	1	100.0 %	0	0
Spalice	2010	13	6.7	1–14 years	L + E	12	92.3 %	78	13	100.0 %	13	100.0 %	0	1
Bilginer	2009	29	10.7	3–21 years	TL ± AHC	?	?	52	27	93.1 %	29	100.0 %	0	?
Lee	2009	22	12.4	3–18 years	L ± L	22	100.0 %	44	20	90.9 %	22	100.0 %	0	1
Minkin	2008	24	8.9	1–15 years	L	21	87.5 %	80	20	83.3 %	24	100.0 %	0	9
Chan	2006	3	11.0	8–14 years	L, L + E	3	100.0 %	104	2	66.7 %	3	100.0 %	0	1
Sandberg	2005	18	9.6	1 month–13 years	L ± map	18	100.0 %	19	18	100.0 %	18	100.0 %	0	1
Cataltepe	2005	14	11.0	4–18 years	L, L + E	12	85.7 %	33	12	85.7 %	14	100.0 %	0	?
Nolan	2004	26	10.0	4–18 years	?	11	42.3 %	52	16	61.5 %	25	96.2 %	0	?
Fernandez	2003	14	10.1	3–18 years	L, L + E	14	100.0 %	87.1	12	85.7 %	13	92.9 %	0	?
Lee	2000	10	9.3	2–15 years	L, L + E	10	100.0 %	40.3	10	100.0 %	10	100.0 %	0	2
Khajavi	1999	7	10.1	4–19 years	L, L + L	6	85.7 %	33	5	71.4 %	7	100.0 %	0	?
		185	9.4	0.5–21 years		130	83.3 %	63.3	159	85.9 %	183	98.9 %	0	13
														11.9 %

In three of the studies, lesionectomies alone were utilized to resect the lesion, with data on the number of total resections available, accounting for 32 patients. Of this number of procedures, 27 yielded a total gross resection (84.4 %) versus 64 of 67 patients in whom lesionectomy was combined with further resection (95.5 %); hence, the odds of an incomplete resection (OR) was 3.49 (95 % confidence interval = 0.78, 15.51, *p* = 0.10).

Over the 13 studies, long-term postoperative freedom from seizures was achieved in 85.9 % of patients, at a mean follow-up of 63.3 months (5.25 years). The percentage of patients achieving seizure freedom in the 12 years from 1994 through 2006 was 81.6 versus 91.3 % in studies published from 2007 onward; however, this difference failed to achieve statistical significance (*t* = 1.20, *df* = 11, *p* = 0.26). Although a moderate direct correlation was noted between the year of paper publication and the percentage seizure freedom, this also failed to achieve statistical significance (*r* = 0.45, *p* = 0.23). On the other hand, the percentage of patients achieving seizure freedom in a given study was both moderately and statistically correlated with the percentage of procedures resulting in gross total resection (*r* = 0.63, *p* = 0.03), but not with mean patient age (*r* = -0.45, *p* = 0.12) or mean duration of follow-up (*r* = 0.20, *p* = 0.51). No correlation at all was noted between the percentage of subjects achieving seizure freedom and any of the three variables *mean duration of seizures prior to surgery* (*r* = −0.007, *p* = 0.98), *percentage of patients with complex partial seizures* (*r* = 0.063, *p* = 0.87), or *percentage of patients with a temporal lobe lesion* (*r* = 0.052, *p* = 0.087).

In the seven studies in which sufficient individual subject data were available, there were 68 gross total resections, resulting in long-term seizure freedom in 62 patients (91.2 %), and 3 subtotal resections, all having Engel stage III outcomes, so that the likelihood of a poor outcome was more than 13 times higher (LR = 13.4; *p* < 0.001) with a subtotal resection. In those same seven studies, 45 of 75 patients underwent a lesionectomy alone and 30 a lesionectomy plus some additional resection, with or without mapping. Of the 45 who underwent lesionectomy alone, 40 (88.9 %) achieve total seizure freedom versus 25 or 30 (83.3 %) among those in whom some additional resection was performed (Pearson *χ* ^2^ = 0.48, *p* = 0.49). By age group, postoperative seizure freedom was achieved in 10 of 11 (90.9 %) of children under age 6, 31 of 35 (88.6 %) children from age 6 up to, but not including 13, and 24 of 29 (82.8 %) of adolescents of 13 years or older, a seemingly downward trend that was not statistically significant (*χ* ^2^ = 0.67, *p* = 0.71). The mean duration of follow-up over these seven studies with individualized data was 40.3 months. Comparing seizure-free rates between patients with a follow-up duration below and above that mean again revealed no significant difference (89.1 vs. 82.2 %, *χ* ^2^ = 0.63, *p* = 0.43).

Similarly, neither duration of seizures (*χ* ^2^ = 2.19, *df* = 3, *p* = 0.54) nor temporal location of the tumor (*χ* ^2^ = 1.44, *df* = 1, *p* = 0.23) was associated with seizure freedom. Since all but one of the patients with seizure-type data had suffered from complex partial seizures, no individualized data analysis for seizure type was performed.

An improvement in seizures was documented in 183 of 185 patients across the ten studies (98.9 %). There were no immediate or late deaths over the course of reported follow-up, and the perioperative complication rate, adjusted for missing data, was just 11.9 %. Postoperative tumor recurrence was reported in two patients, including one whose seizures recurred with tumor recurrence and then improved but did not fully abate following a second resection [30].

## Discussion

DNETs, which typically become manifest during childhood, adolescence, or young adulthood, represent only a small percentage of CNS tumors in either youths or adults [40]. However, these tumors, which typically occur in the temporal lobes [64], are almost always associated with seizures. Together with gangliogliomas and focal cortical dysplasia (FCD), they account for the lion’s share of surgically amenable epileptic brain lesions [43]. Consequently, they comprise a disproportionate percentage of tumor-associated epilepsy cases, especially in children [1, 50, 92, 93, 97, 104, 109]. The reason for the almost ubiquitous presence of seizures with brain tumors like DNETs and gangliogliomas versus much lower rates seen with others, like low-grade gliomas, is not entirely understood [87, 88, 91, 107], although several conjectures have been made, including differential alterations in regional metabolism and pH; immunologic activity; disordered neuronal function; altered vascular supply and permeability; the release of altered tumoral amino acids, proteins, and enzymes; and abnormal protein transport and binding to receptors [1, 14, 87, 91, 93, 107, 114]. Even genetic predispositions for tumor-related seizures have been postulated [8, 107]. To date, all that can be said with confidence is that the cause of tumor-induced seizures is almost certainly multifactorial [114] and beyond the mere physical size of the tumor itself [60].

The risks of surgical resection of an epileptogenic but otherwise “benign” brain tumor, like a DNET, must be weighed against the possible consequences of managing seizures conservatively, due to their potential to inflict significant neurological and cognitive damage, despite favorable rates of survival [28, 66, 67]. In several studies, preoperative cognitive function in pediatric patients with glioneuronal tumors tended to be low average to average and associated with a variety of cognitive deficits, including problems with speech and memory, and delay to meet developmental milestones [24, 29, 32, 34, 82]. Since DNETs tend to be extremely resistant to AED therapy, pharmaceutical control of seizures typically is incomplete [9, 17, 19, 58, 73, 80]. This places patients at risk for continued brain injury and worsening neurocognitive function, prompting many authors to argue for surgical resection of the lesions as soon as possible in most cases [10, 11, 19, 34, 37, 47, 51, 99, 113, 115]. Among the various specific arguments given for early surgical resection of glioneuronal tumors, including DNETS, are the optimization of seizure control [21, 75, 89, 105, 113, 115, 116]; the optimization of brain development [10, 11, 17, 19]; avoiding or at least minimizing the long-term risks of AEDs [22, 53, 66, 68, 96, 107], especially in children and those who may require chemotherapy to control tumor growth [49]; and reducing the risk, albeit low, of later, catastrophic malignant change [2, 20, 41, 61, 71, 72, 81].

Brain surgery is certainly not without its own potential consequences and risks, however, including the prospect of postoperative mortality and worsening seizures and of immediate postoperative neurological deficits and cognitive decline. In their retrospective review of 223 patients 19 years old and under who underwent a combined 229 surgical resections of non-epileptogenic brain tumors, Hardesty et al. identified an incidence of new, postoperative seizures of 7.4 % [42]. On the other hand, almost all were single events that resolved without the need for long-term anti-epileptic drugs (AEDs). Supratentorial tumors, patient age less than 2 years, and the presence of significant postoperative hyponatremia were independent risk factors for new seizures [42]. Presumably, then, these same risk factors of young age and postoperative serum sodium imbalance would at least slightly predispose patients with epileptogenic tumors to having continued or worsened seizures postoperatively. However, worsened seizures postoperatively were reported uncommonly in our review of 13 published studies/series, and the vast majority was transient and controlled prior to hospital discharge. Moreover, Steinbok et al. [99], in their retrospective analysis of 116 pediatric patients under age 3 drawn from eight centers across Canada (mean age at first surgery 15.8 months; range 1–35 months), identified only one surgical death. The most common surgical complications over 151 operations were infection (17) and aseptic meningitis (13). Moreover, more than 1 year postoperatively, 72 (67.3 %) were seizure free and more than 90 % significantly improved. In addition, cognitive development improved in 55.3 % postoperatively.

In the medical literature, the rate of seizure freedom in series with either adults alone or adults plus children has varied widely for DNETs, from as low as 52.4 and 53.3 % [33, 83] to as high as 90 and 100 % [89, 58]. In the current review, we chose to look exclusively at DNETs operated upon during the pediatric years, with one patient aged 21 and all others 19 or younger, to as young as 5 months of age. Overall, in this age group, long-term freedom from seizures was achieved in almost 86 % (85.9 %) and some improvement in 99 % (all but two of 185 patients). Moreover, there were no deaths, and the rate of postoperative complications, the vast majority transient neurological deficits, was only 12 %. The only variable either correlated with seizure freedom rate at a study mean level or associated with seizure freedom at an individual level was degree of tumor resection, with subtotal resections virtually always associated with either the persistence or recurrence of seizures. Patient age was not associated, and neither was whether or not lesionectomy alone or lesionectomy plus some additional resection was performed. These results are congruent with those of a recently published retrospective analysis of 29 children undergoing resection of glioneuronal tumors, in which the rate of seizure freedom 12 months after surgery was 94 % in those in whom gross total resection was achieved versus just 54 % in those in which it was not (*p* < 0.05) [80]; unfortunately, only 13 of the patients were determined to have a DNET, versus 16 with a ganglioglioma, limiting comparisons against our own results. With even more limitations, our results are also consistent with a larger series of 332 patients (mean age 39.3 years, range 16–95) with low-grade gliomas who underwent operative resections for a variety of tumors, in whom seizure control again was far more likely to be achieved after gross total resection than after subtotal resection or biopsy alone (odds ratio 16, 95 % confidence interval 2.2–124, *p* = 0.0064) [18]. One further characteristic that hampers these afore-mentioned studies to some degree is the disproportionate number of complete to incomplete resections. The same is not true of the one series of 26 pediatric patients with DNETs reported by Nolan et al., in which the distribution of gross complete to incomplete resections was fairly evenly split (12 complete, 14 incomplete resections) [73]; in this series, all nine children who had no detectable tumor on postoperative imaging were seizure free at 12 months, with only one relapsing at final follow-up, versus an approximately 50 % rate of seizure freedom in the remainder (*p* = 0.02).

Given that completeness of tumor resection seems to be a determinant of seizure outcomes, the question arises: is it better to do more than just a simple lesionectomy, either via brain mapping to detect epileptogenic foci apart from the tumor itself, or more extensive resections? Over the years, attempts have repeatedly been made to optimize the resection of epileptogenic lesions, both by better delineating their margins and by enhancing the identification of extra-tumoral epileptogenic tissue, using intraoperative tools like electrocorticography (ECoG) to identify potential seizure-inducing tissue irregularities like FCD [4, 7, 28, 35, 46, 58, 75, 89, 111, 112]. This has led to considerable speculation with respect to the relative benefits and safety of performing epilepsy surgery rather than just lesionectomies in patients with tumor-induced seizures [62], even though surgeons have been utilizing additional surgical steps like lobectomy, amygdalohippocampectomy, and, in extreme cases, hemispherotomy for decades [5, 9, 10, 15, 35, 45, 48, 51, 55, 75, 77, 80, 89, 97, 103, 105]. To date, almost no direct empirical comparisons have been undertaken. In perhaps the most methodologically sound study, Gelinas et al. retrospectively compared 34 patients who underwent ECoG-aided epilepsy surgery and 33 patients who had undergone simple lesionectomy without ECoG, all between the ages of 3 months and 16 years, in Vancouver, Canada [35]. One year following surgery, roughly 80 % of patients in each group were seizure free. However, long-term data trended toward improved seizure freedom in patients in the ECoG group, with 79 versus 61 % patients still seizure free at a mean 5.8 years of follow-up (*p* = 0.08). The investigators also noted no increase in neurological morbidity among patients who had undergone the more extensive ECoG-guided cortical resection and that these patients were less likely to require repeat epilepsy surgery [35]. In another smaller retrospective analysis reported by Chan et al. [17], whereas 10 of 12 pediatric DNET patients undergoing a temporal lobectomy achieved seizure freedom, such freedom was achieved in only two of six who had a lesionectomy alone, a difference that, using Pearson *χ* ^2^ analysis, is statistically significant (*χ* ^2^ = 4.5, *p* = 0.03) despite the small numbers. These two studies aside, how effective such tools and approaches are in terms of seizure outcomes, especially in children, remains largely unstudied and, hence, unclear.

The question therefore should no longer be whether or not surgery is indicated in children with an epileptogenic neuroglial tumor like DNET or ganglioglioma, or even at what age such surgery begins to be safe [99, 69], but how surgery should be performed and how aggressive one should be to remove all tumor and/or epileptogenic tissues. In some children, because of the location of the tumor, both in terms of accessibility and proximity to high-function areas of the brain, the child’s overall health status, and perhaps other issues as well, total resection is infeasible. However, even in patients in whom only partial resection was achieved, long-term seizure-free rates have exceeded 50 % [73, 80]. More importantly, in this review of 185 pediatric DNET cases spanning 13 studies and two decades, only two patients failed to improve, and there were no perioperative deaths. In addition, in patients in whom seizure control is initially attained but then lost, repeat surgery appears to be of value. For example, among 106 children (mean age 13.5 years at surgery) who underwent temporal lobe resections for either low-grade tumor or vascular-anomaly-induced epilepsy at The Hospital for Sick Children in Toronto, Canada, between 1983 and 2003, 12 ultimately required a second temporal lobe procedure for intractable recurrent seizures; of these, seven returned to a seizure-free state [6].

Our analysis has admitted limitations, starting with the non-random nature of patient selection which, technically, prohibits the use of certain statistical tests. Note also that the wide range in sample sizes generates weighting issues, in that a report with a single patient was treated statistically the same as series with more than 20. It was for this reason that we attempted to identify as much individual subject data as possible. In fact, this resulted in almost 100 cases being available for analysis, among whom the same association between completeness of resection and long-term seizure freedom was apparent, albeit merely approaching statistical significance. Moreover, we do not claim that our results are empirically definitive; they merely illustrate the inadequacy of current series, all too small to allow for most of the statistical manipulations that we have attempted, and the need for further research, preferably across multiple centers to allow for more adequate patient numbers.

## Conclusions

From this review of 13 studies on DNET resections in children and adolescents, it is clear that surgical resection of the lesion is effective at improving seizures in almost all patients and at achieving long-term seizure freedom in the vast majority. Surgical resection of DNETs also appears to be very safe in children, in terms of both mortality and long-term surgery-related morbidity. Total resection of the lesion appears to be the best predictor of seizure freedom. However, data are lacking on whether this translates into more extensive procedures—like brain mapping and partial lobectomy—being any more effective than simple lesionectomy alone. Further prospective research, preferably involving multiple centers to generate more adequate subject numbers, is clearly indicated.
